# Multiple Pathways Regulate Minisatellite Stability During Stationary Phase in Yeast

**DOI:** 10.1534/g3.112.003673

**Published:** 2012-10-01

**Authors:** Maire K. Kelly, Laura Brosnan, Peter A. Jauert, Maitreya J. Dunham, David T. Kirkpatrick

**Affiliations:** * Department of Genetics, Cell Biology and Development, University of Minnesota, Minneapolis, Minnesota 55455; † Department of Genome Sciences, University of Washington, Seattle, Washington 98195

**Keywords:** DNA stability, stationary phase, G_0_, quiescence

## Abstract

Alterations in minisatellite DNA repeat tracts in humans have been correlated with a number of serious disorders, including cancer. Despite their importance for human health, the genetic factors that influence minisatellite stability are not well understood. Previously, we identified mutations in the *Saccharomyces cerevisiae* zinc homeostasis genes *ZRT1* and *ZAP1* that significantly increase the frequency of minisatellite alteration specifically during stationary phase. In this work, we identified mutants of *END3*, *PKC1*, and *RAD27* that increase minisatellite instability during stationary phase. Genetic analysis reveals that these genes, along with *ZRT1* and *ZAP1*, comprise multiple pathways regulating minisatellite stability during stationary phase. Minisatellite alterations generated by perturbation of any of these pathways occur via homologous recombination. We present evidence that suggests formation of ssDNA or ssDNA breaks may play a primary role in stationary phase instability. Finally, we examined the roles of these pathways in the stability of a human minisatellite tract associated with the *HRAS1* oncogene and found that loss of *RAD27*, but not *END3* or *PKC1*, destabilizes the *HRAS1* minisatellite in stationary phase yeast. This result indicates that the genetic control of stationary phase minisatellite stability is dependent on the sequence composition of the minisatellite itself.

Changes in DNA repeat tracts have long been associated with a number of serious human disorders (Mirkin 2007; Richard *et al.* 2008). Expansions in trinucleotide repeats can cause Huntington’s disease, myotonic dystrophy, and spinocerebellar ataxia (Mirkin 2007). Altered alleles of minisatellites (a class of tandem DNA repeats with repeat units 16–100 nucleotides in length) have also been correlated with several cancer subtypes (Jeong *et al.* 2007; Krontiris *et al.* 1993; Wang *et al.* 2003), progressive myoclonus epilepsy (Lafreniere *et al.* 1997), insulin-dependent diabetes mellitus (Kennedy *et al.* 1995), attention-deficit hyperactivity disorder (Yang *et al.* 2007), asthma (Kirkbride *et al.* 2001), and ulcerative colitis (Kyo *et al.* 1999). Unlike trinucleotide repeat stability, which has been well studied (Mirkin 2007; Richard *et al.* 2008), minisatellite repeat tract stability is only beginning to be understood.

Minisatellites are found throughout eukaryotic genomes (Richard *et al.* 2008). Some bind transcription factors to regulate the expression of nearby genes (Green and Krontiris 1993; Trepicchio and Krontiris 1992) or regulate splicing of the transcript (Jeong *et al.* 2007; Kirkbride *et al.* 2001; Kyo *et al.* 1999). In humans, minisatellites undergo frequent tract length alterations and repeat rearrangements during germline formation, as well as relatively rare somatic tract alterations (Buard *et al.* 2000; Jeffreys and Neumann 1997; Jeffreys *et al.* 1994). We previously showed that human *HRAS1* minisatellite alleles integrated into the genome of *Saccharomyces cerevisiae* recapitulates this pattern of stability (Jauert *et al.* 2002), as have other groups (Debrauwere *et al.* 1999), thus allowing yeast to serve as a model organism for studying minisatellite stability.

The yeast genome has a number of native minisatellite sequences, with many clustered near the telomeres [reviewed in Richard and Dujon (2006)], but the stability of most of these repetitive DNA tracts has not been examined systematically. However, some native and a number of introduced minisatellite sequences have been shown to alter during meiosis. Meiotic minisatellite alterations require the recombination-initiated endonuclease Spo11, the large loop repair endonuclease Rad1 (Jauert *et al.* 2002), and the *RAD50* recombination protein (Debrauwere *et al.* 1999). During mitotic growth, minisatellites are relatively stable but can be destabilized by loss of the flap endonucleases Rad27 and Dna2, yeast PCNA Pol30, DNA polymerase Pol3, or DNA helicase Pif1 (Kokoska *et al.* 1999; Lopes *et al.* 2002; Maleki *et al.* 2002; Ribeyre *et al.* 2009).

We recently demonstrated that minisatellite stability is controlled in stationary phase cells. We used a colony color assay system for assessing minisatellite stability (Kelly *et al.* 2007) that is unique in its ability to distinguish mitotic alterations, which are seen as sectored colonies, from minisatellite alterations that occur during stationary phase, which are seen as white microcolonies forming on the surface of the main colony; we call this novel color segregation phenotype “blebbing.” Using this system, we found that mutations in the zinc homeostasis genes *ZRT1* and *ZAP1* lead to increased minisatellite alterations during stationary phase (Kelly *et al.* 2007), specifically in the truly quiescent subset of G_0_ cells in stationary phase (Kelly *et al.* 2011). Further, these alterations require homologous recombination and also occur in a human-derived minisatellite associated with the *HRAS1* oncogene (Kelly *et al.* 2011).

In this study, we describe additional mutants that show increased minisatellite alterations during stationary phase. Mutations in the endocytosis gene *END3*, the essential protein kinase encoded by *PKC1*, or the flap endonuclease gene *RAD27* all lead to an increase in stationary phase minisatellite tract expansions or contractions, and these tract alterations are dependent on recombination factors. Genetic analysis indicates that multiple pathways regulate minisatellite stability during stationary phase. A common factor affecting minisatellite stability in these mutants may be an effect on ssDNA formation. Finally, we examine the stability of a human disease-associated minisatellite in these mutants.

## Materials and Methods

### Media, plasmids, and strains

Standard media (Guthrie and Fink 1991) was used, except for YPD + G418, which was made by the addition of 200 mg/L of G418 sulfate (geneticin) to standard YPD solid media. Sporulation and tetrad dissection protocols used in this study have been previously reported (Jauert *et al.* 2002).

All *S. cerevisiae* strains examined in this study (Table 1) are derived from EAS28 (Sia *et al.* 2001), a W303 derivative closely related to S288c (Schacherer *et al.* 2007). Strains whose construction is not reported here have been previously described (Kelly *et al.* 2007). Two minisatellite alleles were used in this study: *ade2-min3* (Kelly *et al.* 2007), which is an artificial minisatellite initially used to examine mismatch repair in yeast (Sia *et al.* 1997), and *ade2-h7.5*, (Kelly *et al.* 2011), which was derived from a minisatellite associated with the human *HRAS1* gene which was inserted into the *HIS4* locus on chromosome III as described (Jauert *et al.* 2002). Strains DTK1088 and DTK1266, bearing deletions of *END3*, were constructed by PCR of the *end3*Δ::*KAN* cassette using DNA from the *END3* deletion Saccharomyces Deletion Consortium (SDC) strain and primers 28278222 and 28278223. The *irc10*Δ::*KAN* strain DTK1091 was constructed in similar fashion, using primers 28234947 and 28234948 and DNA from the corresponding SDC strain. Strains DTK1012 and DTK1225, bearing deletions of *RAD27*, were also generated by PCR with the DNA of a *rad27*Δ::*KAN* SDC strain using primers 23094593 and 23094594. DTK1379, bearing a deletion of *PTC1*, was constructed by PCR with DNA from the appropriate SDC strain using primers 44775228 and 44775229. DTK1360, bearing a deletion of *ETR1*, and DTK1361, bearing a deletion of *POR1*, were constructed transformation of DTK271 with PCR products as above using primers 37616572 and 37616573 and primers 37616574 and 37616575, respectively. All PCRs generated a product containing the KANMX4 geneticin resistance gene flanked with 5′ and 3′ homology to the targeted gene. These cassettes were transformed into the parental strains and integration events were selected on YPD + G418. All transformants were verified by PCR.

**Table 1:** Yeast strains used in this study

Strain	Relevant Genotype	Construction Details (Reference)
EAS28	Wild-type	*MAT*a *his7-2 trp1-289 ura3-52* (Sia *et al.* 2001)
DTK260	*leu2*::*HisG*	EAS28 with pNKY85 (Kelly *et al.* 2007)
DTK264	*ade2-min3*	DTK260 with pDTK123 (Kelly *et al.* 2007)
DTK271	*ade2-min3*, *MAT*α	DTK264 with pGal-HO (Herskowitz and Jensen 1991)
DTK284	*ade2-min3*, *arg8*::*HisG*	DTK264 with pDS27
DTK904	*ade2-min3*, *zrt1*::*LEU2*	DTK284 with *zrt1*::*LEU2**^a^* (Kelly *et al.* 2007)
DTK1012	*ade2-min3*, *zrt1*::*LEU2*, *rad27*::*KAN*	DTK904 with *rad27*::*KAN**^a^*
DTK1056	*ade2-min3*, *rad50*::*KAN*	DTK271 with *rad50*::*KAN* (Kelly *et al.* 2007)
DTK1074	*ade2-min3*, *rad51*::*KAN*	DTK271 with *rad51*::*KAN**^a^*
DTK1088	*ade2-min3*, *end3*::*KAN*	DTK271 with *end3*::*KAN**^a^*
DTK1091	*ade2-min3*, *irc10*::*KAN*	DTK271 with *irc10*::*KAN**^a^*
DTK1174	*ade2-min3*, *zrt1*::*LEU*, *end3*::*KAN*	DTK904 x DTK1088, isolated spore
DTK1185	*ade2-min3*, *end3-1*, *ras2*::*KAN*	Y797 with *ras2*::*KAN**^a^*
DTK1186	*ade2-min3*, *ras2*::*LEU2*	DTK271 with *ras2*::*LEU2**^a^*
DTK1187	*ade2-min3*, *end3*::*KAN*, *ras2*::*KAN*	DTK1088 x DTK1186, isolated spore
DTK1188	*ade2-h7.5*	DTK260 with pKK055, FOA^R^ isolate
DNY101	*rad52*::*URA3*	(Nag and Petes 1993)
DTK1199	*ade2-min3*, *rad27*::*KAN*	DTK271 x DTK1012, isolated spore
DTK1205	*jnm1*::*KAN*	Spore isolated from Yeast Deletion Consortium strain dissection
DTK1218	*ade2-min3*, *end3-1*, *rad27*::*KAN*	Y797 x DTK1199, isolated spore
DTK1224	*ade2-min3*, *rad27*::*KAN*, *rad52*::*URA3*	DTK1199 x DTK1253, isolated spore
DTK1225	*ade2-h7.5*, *rad27*::*KAN*	DTK1188 with *rad27*::*KAN**^a^*
DTK1227	*ade2-min3*, *end3-1*, *rad52*::*URA3*	Y797 x DTK1191, isolated spore
DTK1247	*ade2-min3*, *jnm1*::*KAN*	DTK271 x DTK1205, isolated spore
DTK1253	*ade2-min3*, *rad52*::*URA3*	DTK1191 x DTK284, isolated spore
DTK1266	*ade2-h7.5*, *end3*::*KAN*	DTK1188 with *end3*::*KAN**^a^*
YKH27	*pkc1-4*	(Huang and Symington 1995)
DTK1279	*ade2-min3*, *pkc1-4*	DTK271 x YKH27, isolated spore
DTK1288	*ade2-min3*, *zrt1*::*LEU2*, *pkc1-4*	DTK904 x DTK1279, isolated spore
DTK1289	*ade2-min3*, *rad50*::*KAN*, *rad52*::*URA3*	DTK1268 x DTK284, isolated spore
DTK1290	*ade2-min3*, *rad51*::*KAN*, *rad52*::*URA3*	DTK1269 x DTK284, isolated spore
DTK1293	*ade2-min3*, *end3*::*KAN*, *pkc1-4*	DTK1088 x DTK1279, isolated spore
DTK1294	*ade2-min3*, *rad27*::*KAN*, *pkc1-4*	DTK1199 x DTK1279, isolated spore
DTK1316	*ade2-min3*, *dnl4*::*KAN*	DTK271 with *dnl4*::*KAN**^a^*
DTK1346	*ade2-min3*, *pkc1-4*, *rad52*::*URA3*	DTK1191 x DTK1279, isolated spore
DTK1357	*ade2-min3*, *pkc1-4*, *rad50*::*KAN*, *rad52*::*URA3*	DTK1056 x DTK1346, isolated spore
DTK1358	*ade2-min3*, *pkc1-4*, *rad51*::*KAN*, *rad52*::*URA3*	DTK1074 x DTK1346, isolated spore
DTK1360	*ade2-min3*, *etr1*::*KAN*	DTK271 with *etr1*::*KAN**^a^*
DTK1361	*ade2-min3*, *por1*::*KAN*	DTK271 with *por1*::*KAN**^a^*
DTK1362	*ade2-min3*, *pkc1-4*, *rad50*::*KAN*	DTK271 x DTK1357, isolated spore
DTK1363	*ade2-min3*, *pkc1-4*, *rad51*::*KAN*	DTK271 x DTK1358, isolated spore
DTK1364	*ade2-min3*, *rad27*::*KAN*, *etr1*::*KAN*	DTK1199 x DTK1360, isolated spore
DTK1367	*ade2-min3*, *pkc1-4*, *etr1*::*KAN*	DTK1279 x DTK1360, isolated spore
DTK1368	*ade2-min3*, *pkc1-4*, *por1*::*KAN*	DTK1279 x DTK1361, isolated spore
DTK1370	*ade2-min3*, *rad27*::*KAN*, *por1*::*KAN*	DTK1199 x DTK1361, isolated spore
DTK1371	*ade2-min3*, *end3*::*KAN*, *etr1*::*KAN*	DTK1088 x DTK1360, isolated spore
DTK1372	*ade2-min3*, *end3*::*KAN*, *por1*::*KAN*	DTK1088 x DTK1361, isolated spore
DTK1373	*ade2-h7.5*, *end3*::*KAN*	DTK1188 with *end3*::*KAN**^a^*
DTK1375	*ade2-h7.5*, *pkc1-4*	DTK1188 x DTK1279, isolated spore
DTK1379	*ade2-min3*, *zrt1*::*LEU*, *ptc1*::*KAN*	DTK904 with *ptc1*::*KAN**^a^*
DTK1386	*ade2-min3*, *pkc1-4*, *ptc1*::*KAN*	DTK1279 x DTK1379, isolated spore
DTK1408	*ade2-min3*, *pkc1-4*, *dnl4*::*KAN*	DTK1279 x DTK1316, isolated spore

a Strain was made using a PCR-generated construct.

Strain DTK1247, bearing a deletion of *JNM1*, was constructed by mating. The *jnm1*Δ::*KAN* diploid SDC strain was sporulated and dissected, and a haploid spore (DTK1205) of the desired mating type was isolated. DTK1205 was mated to DTK271, and the resulting diploid was sporulated and dissected. An *ade2-min3 jnm1*Δ::*KAN* spore was isolated by color and ability to survive on YPD + G418 media. This spore isolate was backcrossed twice to DTK271, and each time an *ade2-min3 jnm1*Δ::*KAN* spore isolate was identified as described above. DTK1247 is the spore isolate of the final backcross. DTK1279, bearing the temperature-sensitive point mutation *pkc1-4*, was also generated by mating. The *pkc1-4* strain YKH27 (Huang and Symington 1994) was crossed to DTK271, and the resulting diploid was sporulated and dissected. An *ade2-min3 pkc1-4* spore was isolated by color and temperature sensitivity at 37°. This spore isolate was backcrossed to DTK271 twice to generate DTK1279, the final *ade2-min3 pkc1-4* spore isolate.

### Whole-genome hybridization

Whole-genome hybridization of DTK271 and Y797 DNA and analysis of the resulting profiles were conducted as previously described (Gresham *et al.* 2006).

### Minisatellite tract length analysis by PCR

White cells from independent blebs were picked with sterile toothpicks, patched on YPD, and incubated at 30° overnight. Whole-cell PCR across the *ade2-min3* minisatellite tract was conducted for each independent bleb isolate, plus the wild-type *ade2-min3* strain using primers 43901571 and 43901572. Five random PCR products were sequenced using primers 17339862 and 17339863 to confirm that changes in size compared with wild-type *ade2-min3* were due to changes in the minisatellite repeat tract.

### Flow cytometry

Flow cytometry was conducted as previously reported (Gourlay and Ayscough 2006), with minor alterations. The wild-type parent (DTK271), *end3*Δ (DTK1088), and *end3Δ ras2*Δ (DTK1187) strains were grown at 30° for 48 hr in 5ml of YPD in the presence of 5μg/ml 2′,7′-dichlorodihydrofluorescein (H_2_DCF-DA; Molecular Probes). Cells were sonicated briefly prior to analysis, and fluorescence was analyzed on a FACSCalibur benchtop cytometer (BD Biosciences). Data were analyzed using CellQuest Pro (BD Biosciences).

### Quantification of blebbing

The frequency of bleb formation on individual colonies was determined using previously described protocols (Kelly *et al.* 2011). Colonies were grown at 30° for 3 days and then incubated at room temperature for 4 days. Colonies were photographed, and the number of blebs on the colony surface were counted. At least 100 colonies were examined for each strain, and each strain was assayed three times independently. Subsequently, the mean number of blebs per colony was calculated and the 95% confidence interval for the mean was determined.

### Primers

The following primers were used in this study:

Primer 28278222 (*End3F*): GAGTTAGTGGGTATTGGAAAGGC
Primer 28278223 (*End3R*): CCACACCGTTACTGGATAGA
Primer 28234947 (*Irc10F*): TGAGTGGACACAGAAAACGC
Primer 28234948 (*Irc10R*): CAGTACAGTTTCGCTAAGTAAGG
Primer 23094593 (*Rad27F*): GCGTCCCATCGCGCAAATGAAG
Primer 23094594 (*Rad27R*): TCCACGTTCAAGTTCCCAGAAA
Primer 44775228 (*Ptc1F*): ACAGACCCCAAACACAACAAG
Primer 44775229 (*Ptc1R*): CCTCATTCGTCATGTGAGAGATGC
Primer 43901571 (*ade2-min3F*): GGTGCGTAAAATCGTTGGATCTC
Primer 43901572 (*ade2-min3R*): GCTCAATCTCAATCGTTAGCAC
Primer 17339862 (*ade2-min3 seqF*): CGGACAAAACAATCAAGTATGG
Primer 17339863 (*ade2-min3 seqR*): ATGTTGAGCCTGTTTGCTG
Primer 37616573 (Etr1F): TGTACCCAGGGGTGGTTTCCAT
Primer 37616572 (Etr1R): TTGAAGGGTCGACGTCCCCTTTTA
Primer 37616575 (Por1F): CCAATCAAACACCGCCATTTCG
Primer 37616574 (Por1R): TTCTCACTGCCAAGCAACCA

## Results

**Figure 1:**
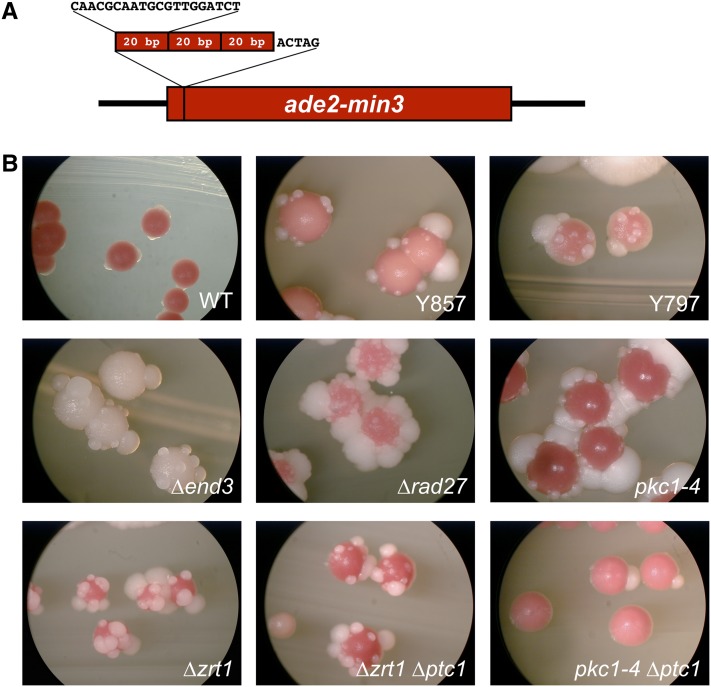
The color-based *ade2-min3* reporter was used to identify factors that regulate minisatellite stability. (A) The *ade2-min3* allele. Three 20 bp minisatellite repeats plus one additional bp were inserted into the *ADE2* gene at the *Xba*I site. Duplication of the 4 nt *Xba*I overhang yielded a 65 bp insertion, resulting in a frameshift that disrupts *ADE2*. Loss of one 20 bp repeat unit, or gain of two repeat units, restores *ADE2* to the correct reading frame. (B) Red/white color segregation in *ade2-min3* strains. Strains were grown at 30° for 3 days, and then at room temperature for 4 days. The *pkc1-4* mutant was grown at the semi-permissive temperature of 35° for 7 days. The wild-type *ade2-min3* parent is DTK271. Y857 and Y797 are UV-generated point mutants of *JNM1* and *END3*, respectively. Construction of the remaining strains is described above: *end3*Δ (DTK1088), *rad27*Δ (DTK1199), *pkc1-4* (DTK1279), *pkc1-4 ptc1*Δ (DTK1386), *zrt1*Δ (DTK904), and *zrt1Δ ptc1*Δ (DTK1379).

We previously described the *ade2-min3* allele, a color-based reporter of minisatellite stability in *S. cerevisiae* (Kelly *et al.* 2007). This allele is composed of three tandem 20 base pair minisatellite repeats, plus 1 nucleotide, integrated into the *ADE2* gene at an *Xba*I site (Figure 1A). With duplication of the 4 nt *Xba*I overhangs, this insertion shifts the reading frame of *ADE2*, disrupts adenine biosynthesis, and results in a red colony color. However, loss of one 20 bp minisatellite repeat unit restores the correct *ADE2* reading frame, adenine production, and white color. If such minisatellite alterations occur during growth of the colony, a white sector forms within the red colony. If minisatellite alterations occur after growth of the colony has arrested, white papillations will form on the surface of the red colony. We designated this novel color segregation phenotype “blebbing.”.

### Identification and characterization of blebbing mutants

We utilized the *ade2-min3* reporter in a screen for mutants that increased the frequency of minisatellite alterations (Kelly *et al.* 2007). Four complementation groups with a blebbing phenotype were identified; the frequency of alleles in each group indicates that the screen was not saturated and that there are likely to be other genes that may be mutated to give a blebbing phenotype. The genes mutated in two of the four complementation groups were *ZRT1* and *ZAP1*. While we previously described the cloning and characterization of these genes and their role in stationary phase minisatellite stability (Kelly *et al.* 2007, 2011), the mutations in the complementation groups represented by strains Y797 and Y857 (Figure 1B), each composed of one allele, remained to be cloned. These mutant strains were crossed to the parental DTK271 strain; the resulting diploids did not bleb, indicating that the mutations in Y797 and Y857 are recessive. We sporulated the diploids from these backcrosses, dissected tetrads, and observed the segregation of the blebbing phenotype. The resulting tetrads exhibited 2:2 segregation of the blebbing phenotype, indicating that a single mutation is responsible for blebbing in Y797 and Y857. Y797 is temperature sensitive at 37°, and the temperature sensitivity is tightly linked to the Y797 blebbing phenotype.

To clone the mutation responsible for blebbing in Y857, we used our previously described yeast genomic library on a geneticin-resistance plasmid vector (Jauert *et al.* 2005). Library plasmids were rescued from Y857 transformants that exhibited the parental (non-blebbing) phenotype, and the genomic insert was sequenced to identify candidate genes. The complementing plasmid 2a1b carries nucleotides 846000 to 863501 of chromosome XII; one of the genes in this interval, *JNM1*, encodes a dynactin subunit. Sequencing of *JNM1* in Y857 revealed a C to T substitution that changes amino acid 133 from a glutamine to a stop codon. Deletion of *JNM1* in the *ade2-min3* background resulted in a blebbing phenotype, but the phenotype was highly variable and prevented further analysis.

Identification of the mutated gene in the Y797 complementation group was difficult. Initially we attempted to clone the mutation by complementation using the same protocols we used with Y857. However, Y797 did not survive the heat shock step of the transformation protocol, and it was also recalcitrant to electroporation or spheroplast transformation, presumably due to the temperature sensitivity that is linked to the blebbing phenotype. Attempts at cloning by complementation using low-temperature variations of the transformation protocols were also unproductive. Finally, we were unsuccessful in attempts to map the location of the mutation using mapping strains (Wakem and Sherman 1990). Therefore, we utilized whole-genome hybridization (Gresham *et al.* 2006) to identify the mutation responsible for blebbing in Y797. Briefly, Y797 was backcrossed to the parental strain DTK271 three times to limit the amount of polymorphism between these strains. Genomic DNA was then isolated from Y797 and DTK271 and hybridized to two separate yeast genomic tiling arrays. These microarrays are specially designed with multiple overlapping probes to detect single nucleotide polymorphisms (SNP) between the reference strain used to construct the microarray and the query strain. The SNP profiles of DTK271 and Y797 were then compared with highlight differences between the strains. Two such polymorphisms were identified in Y797, and the genes at the indicated locations were sequenced. A single nucleotide deletion in the *END3* gene changed amino acid 174 from a leucine to a stop codon, and a point mutation in the *IRC10* gene changed amino acid 138 from a lysine to a stop codon. Deletion of *IRC10* in the *ade2-min3* background did not result in a blebbing phenotype, but an *ade2-min3 end3*Δ mutant blebbed and was temperature sensitive at 37° (Figure 1B and data not shown). The diploid product of a cross between Y797 and an *ade2-min3 end3*Δ strain exhibits a blebbing phenotype. Finally, we monitored formation of white cells in liquid culture using a time course protocol previously performed with the *ade2-min3 zrt1*Δ strain (Kelly *et al.* 2007). Culture growth was monitored by OD_600_ at intervals, and aliquots were concurrently diluted and plated on rich media to determine the frequency of minisatellite alterations by relative number of white Ade^+^ colony-forming units (CFU) for the parental strain (DTK271), Y797, and an *end3*Δ mutant (DTK1088). Strains reached stationary phase after 96 hr at room temperature; at 120 hr we observed the first significant increase in the percentage of white CFUs in both of the *end3* mutants compared with the parental strain (*P* < 0.02 for both, using Student *t*-test). These results confirm that, as with *ZRT1*, blebbing in an *end3* mutant is caused by minisatellite alterations occurring specifically during stationary phase.

We examined mutants that were previously shown to affect minisatellite stability in actively growing cells, and we identified two additional genes that affect minisatellite stability in stationary phase. Deletion of *RAD27*, which encodes the yeast FEN-1 flap endonuclease (Liu *et al.* 2004), has been linked to minisatellite instability in actively growing cells (Lopes *et al.* 2002; Maleki *et al.* 2002). Loss of *RAD27* results in a blebbing phenotype in the *ade2-min3* strain background (Figure 1B), demonstrating a role for *RAD27* in stationary phase cells. Sectors can also be observed in some *ade2-min3 rad27*Δ colonies, in agreement with prior reports that *RAD27* regulates minisatellite stability during mitotic growth (Lopes *et al.* 2002; Maleki *et al.* 2002). A temperature-sensitive allele of *PKC1*, an essential protein kinase, previously was shown to have a hyper-recombination phenotype using a direct-repeat recombination assay (Huang and Symington 1994). Lorraine Symington provided us with the *pkc1-4* temperature-sensitive point mutant, as the colony morphology of the strain was reminiscent of the blebbing phenotype of our *zrt1*Δ mutants. We crossed this mutation into our *ade2-min3* background, using the temperature sensitivity to track the *pkc1-4* allele, and we found that the *pkc1-4 ade2-min3* strain blebs at 35° (Figure 1B).

To characterize the minisatellite alterations that result in white Ade^+^ blebs in each of our blebbing mutants, we conducted a PCR analysis of the *ade2-min3* minisatellite tract length from white cells isolated from individual blebs. At least 100 independent PCR products were examined from each of the *end3*Δ, *rad27*Δ, and *pkc1-4* mutant strains. Bleb formation in both the *end3*Δ and *pkc1-4* mutants was exclusively caused by loss of one *ade2-min3* minisatellite repeat unit, as described for the *zrt1*Δ mutant (Kelly *et al.* 2007). In contrast, loss of one minisatellite repeat was responsible for 51% (52/102) of blebs formed in the *rad27*Δ mutant, while gain of two minisatellite repeats accounted for the remaining 49% (50/102) of blebs.

Stationary phase *S. cerevisiae* cells can be in a truly quiescent state or in a nonquiescent, very slowly dividing, state (Allen *et al.* 2006). These states can be distinguished genetically: loss of *ETR1*, encoding a thiolester reductase, prevents quiescent cells from reentering the cell cycle, whereas loss of *POR1*, encoding a mitochondrial porin, has a similar effect on nonquiescent cells. We previously demonstrated that a *zrt1Δ etr1*Δ mutant does not bleb [0.09 blebs/colony; Kelly *et al.* 2011)], indicating that the minisatellite alterations occurring in the *zrt1*Δ mutant arise in quiescent cells. We performed a similar analysis on *rad27*Δ, *end3*Δ, and *pkc1-4* mutants. Alterations in the *rad27*Δ mutant (Table 2, 25.4 blebs/colony) occur primarily in quiescent cells: loss of *ETR1* has a significant effect (0.5 blebs/colony), whereas loss of *POR1* has only a small effect (20.0 blebs/colony). Similarly, in *end3*Δ cells (4.8 blebs/colony), loss of *ETR1* (0.4 blebs/colony) has a much greater effect than does loss of *POR1* (2.3 blebs/colony). The *pkc1-4* mutant (11.7 blebs/colony) was harder to evaluate; loss of *ETR1* significantly reduced blebbing (to 0.03 blebs/colony), but loss of *POR1* also had a strong effect (to 1.4 blebs/colony).

**Table 2:** Quantitative analysis of blebbing in double mutant strains

		Second Relevant Genotype			
		WT	*rad27*Δ	*end3*Δ	*pkc1-4**^a^*
First Relevant Genotype	WT	3.7 ± 0.4*^b^*	25.4 ± 1.0	4.8 ± 0.4	11.7 ± 0.7
	*zrt1*Δ	20.6 ± 0.8	32.0 ± 1.2	3.4 ± 0.3	16.3 ± 1.0
	*pkc1-4**^a^*	11.7 ± 0.7	28.5 ± 1.1	2.9 ± 0.3	ND
	*end3-1*	8.5 ± 0.6	29.4 ± 1.9	ND	ND

ND, no data.

a Denotes colonies grown at 35°.

b Mean blebs per colony ± 95% confidence interval.

### Genetic analysis of blebbing mutants

The blebbing phenotypes of the various mutants differed significantly (Figure 1B). We quantified the amount of blebbing in each strain (Table 2) to compare the relative level of minisatellite instability. The *rad27* and *zrt1* mutants exhibited the highest level of blebbing (25.4 and 20.6 blebs/colony, respectively), both significantly above the parental DTK271 strain (3.7 blebs). The *pkc1-4* strain had 11.7 blebs/colony at the 35° restrictive temperature. The two *END3* mutants we evaluated exhibited significant differences: the *end3-1* allele isolated from Y797 had 8.5 blebs/colony, whereas the deletion of *END3* had 4.8. In addition, colonies from the deletion mutant were significantly less red than was the point mutant (Figure 1B). These data indicate that the Y797 *end3-1* mutation is likely a hypomorphic allele rather than a complete loss-of-function allele.

To determine how many independent pathways monitor minisatellite stability during stationary phase, we constructed double mutant strains containing pairwise combinations of *ZRT1*, *END3*, *RAD27*, and *PKC1* mutant alleles (Table 2). Where blebbing was higher in the double mutant than in either of the parental single mutants (as determined by non-overlap of the 95% confidence intervals for the mean), the two genes were considered to potentially participate in separable pathways regulating stationary phase minisatellite stability, whereas if blebbing was lower or not significantly different, the two genes were considered to participate in similar pathways.

Quantification of blebbing revealed that the *rad27Δ zrt1*Δ mutant had an average of 32.0 blebs per colony (Table 2), higher than the *rad27*Δ single mutant (25.4 blebs/colony) or the *zrt1*Δ single mutant (20.6 blebs/colony). This result indicates that *RAD27* and *ZRT1* have at least partially independent roles in monitoring minisatellite stability during stationary phase. Similar results were seen for *rad27*Δ with *END3* or *PKC1* mutants. A *rad27Δ end3-1* strain had 29.6 blebs/colony, higher than the *rad27*Δ (25.4 blebs/colony) or *end3-1* (8.5 blebs) single mutants. Likewise, a *rad27Δ pkc1-4* strain had 28.5 blebs/colony, whereas a *pkc1-4* mutant had 11.7 blebs/colony. Therefore, the *RAD27* protein acts in pathways that are partially, but not fully, distinct from the *END3* and *PKC1* protein pathways. This classification is supported by the alteration types seen in the *rad27*Δ mutant (described above); only the *rad27*Δ mutant exhibited *ade2-min3* tract length increases. No other double mutants exhibited higher blebbing than the parental single mutants, indicating that *END3*, *PKC1*, and *ZRT1* could potentially function in overlapping pathways regulating minisatellite stability during stationary phase.

Minisatellite alterations in a *zrt1*Δ mutant require recombination factors (Kelly *et al.* 2007, 2011). We examined *END3* and *PKC1* mutants to determine the influence recombination factors have in those backgrounds. The majority of homologous recombination in *S. cerevisiae* requires *RAD52* (Coic *et al.* 2008). Deletion of *RAD52* in an *end3-1* strain reduces blebbing to 0.6 blebs/colony from 8.5 blebs/colony (Table 3). Because blebbing in the *end3-1 rad52*Δ double mutant is not significantly different from spontaneous blebbing in the *rad52*Δ single mutant (1.4 blebs/colony) as determined by overlap of the 95% confidence intervals, we conclude that all minisatellite alterations in *END3* mutants occur via *RAD52*-dependent recombination.

**Table 3:** Quantitative analysis of blebbing in strains with deletions of recombination factors

		Second Relevant Genotype						
		WT	*rad50*Δ	*rad51*Δ	*rad52*Δ	*rad51*Δ *rad52*Δ	*rad50*Δ *rad52*Δ	*dnl4*Δ
First Relevant Genotype	WT	3.7 ± 0.4*^a^*	0.9 ± 0.2	2.1 ± 0.3	1.4 ± 0.2	1.7 ± 0.2	0.6 ± 0.2	1.8 ± 0.3
	*pkc1-4**^b^*	11.7 ± 0.7	3.1 ± 0.5	3.9 ± 0.4	5.7 ± 0.6	2.1 ± 0.5	0.4 ± 0.2	9.0 ± 1.0
	*end3-1*	8.5 ± 0.6	ND	ND	0.6 ± 0.2	ND	ND	ND

ND, no data.

a Mean blebs per colony ± 95% confidence interval.

b Denotes strains grown at 35°.

In a *PKC1* mutant strain, loss of *RAD52* only partially reduces blebbing, to 5.7 blebs/colony compared with 11.7 blebs/colony in the parental *pkc1-4* strain (Table 3). Therefore, approximately 50% of minisatellite alterations in a *pkc1-4* mutant occur by *RAD52*-dependent recombination. In *S. cerevisiae*, *RAD52*-independent homologous recombination requires *RAD50* and/or *RAD51*. A *pkc1-4 rad51Δ rad52*Δ triple mutant had 2.1 blebs/colony, a greater reduction in blebbing than the *pkc1-4 rad52*Δ double mutant displayed, indicating that some *RAD52*-independent recombination requires *RAD51* in the *pkc1-4* mutant. The *pkc1-4 rad50Δ rad52*Δ strain exhibited 0.5 blebs/colony, which was not significantly different from the *rad50Δ rad52*Δ double mutant (0.6 blebs/colony), showing that all *RAD52*-independent minisatellite alterations in the *pkc1-4* mutant require *RAD50*. As *RAD50* is required for non-homologous end-joining (NHEJ) as well as recombination, we deleted *DNL4*, which encodes a DNA ligase required for NHEJ, in the *pkc1-4* strain background. The *pkc1-4 dnl4*Δ double mutant displayed an average of 9.0 blebs/colony, not substantially different from the *pkc1-4* parent strain (11.7 blebs/colony).

The results of our recombination mutant analysis provide further differentiation between pathways regulating minisatellite stability during stationary phase. Because mutations in *ZRT1* or *PKC1* generate both *RAD52*-dependent and *RAD52*-independent minisatellite alterations (Kelly *et al.* 2011), their gene products clearly act differently than the *END3* protein, as disruption of *END3* results in only *RAD52*-dependent minisatellite alterations. We could not perform a similar analysis with *RAD27*, as *rad27Δ rad52*Δ mutants are inviable (Symington 1998).

We were next interested in determining whether *ZRT1* and *PKC1* function in the same pathway. It has been shown that *pkc1-4* displays a hyper-recombination phenotype that can be suppressed by loss of the protein phosphatase encoded by *PTC1* (Huang and Symington 1995). *PTC1* is required for TOR signaling in yeast (Gonzalez *et al.* 2009). Deletion of *PTC1* in our *ade2-min3 pkc1-4* mutant suppressed blebbing (Figure 1B). However, loss of *PTC1* in a *zrt1*Δ mutant did not suppress blebbing. The differential effect of the *PTC1* mutation indicates that *ZRT1* and *PKC1* regulate minisatellite stability via differing pathways during stationary phase.

### Reactive oxygen species production in the end3Δ mutant

**Figure 2:**
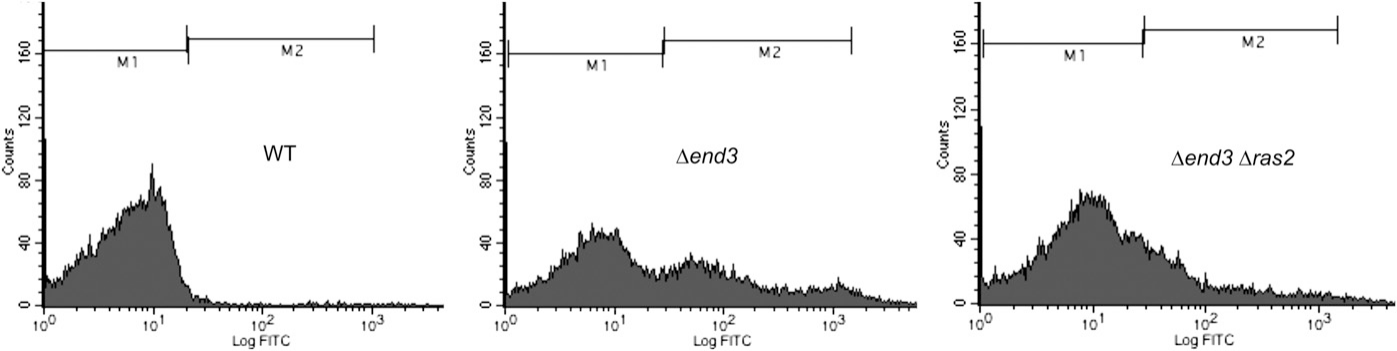
*END3* mutants display *RAS2*-dependent ROS accumulation during stationary phase. ROS accumulation in wild-type, *end3*Δ, and *end3Δ ras2*Δ stationary phase cells was assayed using H_2_DCF-DA by flow cytometry (see *Materials and Methods*). The data from one of three independent assays are displayed on histograms and divided into M1 (low ROS) and M2 (high ROS) populations.

The *RAD27* and *PKC1* proteins have roles that influence genome maintenance (Ayyagari *et al.* 2003; Huang and Symington 1994; Wu and Wang 1999), but *END3*, which encodes a protein involved in endocytosis (Benedetti *et al.* 1994), has no obvious role. However, it has been reported that during stationary phase *END3* mutants produce high levels of reactive oxygen species (ROS) via *RAS2* hyperactivation (Gourlay and Ayscough 2006). ROS can produce a variety of DNA lesions, including DNA single- and double-strand breaks (Jackson and Loeb 2001). To investigate whether elevated ROS may be responsible for blebbing in our *END3* mutant strain, we first assessed ROS production in our strains by staining with H_2_DCF-DA as described (Gourlay and Ayscough 2006). Wild-type, *end3*Δ, and *end3Δ ras2*Δ strains were incubated in rich liquid media in the presence of H_2_DCF-DA for 48 hr, then evaluated by flow cytometry. The *end3*Δ strain displayed elevated levels of ROS staining compared with the wild-type parent (Figure 2). Loss of *RAS2* in the *END3* mutant background reduced but did not completely abolish ROS staining. Similar results were observed when this experiment was repeated with *end3-1* and *end3-1 ras2*Δ strains (data not shown). An *end3-1* mutant displays an average of 8.5 blebs per colony. Blebbing in the *end3-1 ras2*Δ double mutant is reduced to 3.5 blebs/colony. The concurrent drop in ROS staining levels and blebbing in *end3-1 ras2*Δ double mutants suggests that ROS production is possibly linked to minisatellite instability in *END3* mutants.

### Stability of the human HRAS1 minisatellite in RAD27, END3, and PKC1 mutants

**Figure 3:**
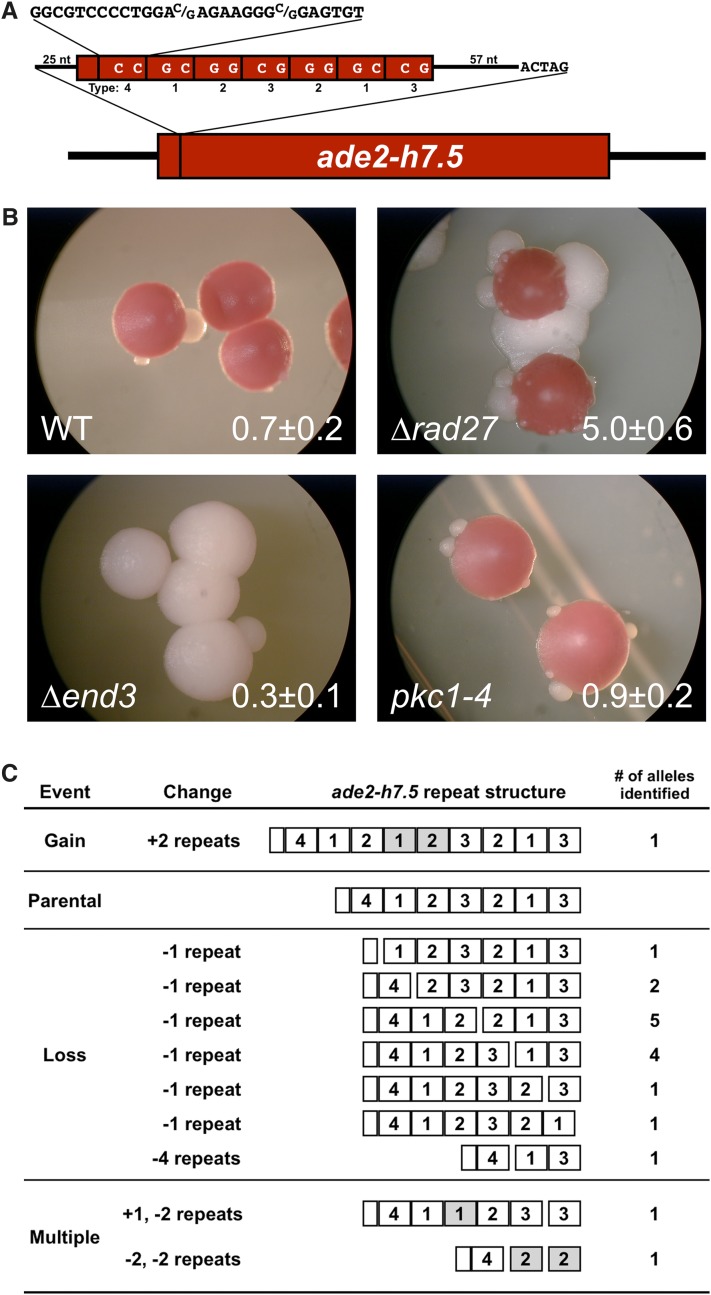
The color-based *ade2-h7.5* reporter was used to identify factors that regulate the stability of the human *HRAS1* minisatellite. (A) The *ade2-h7.5* allele. Seven and one-half repeats of 28 bp derived from the human *HRAS1* minisatellite were inserted into *ADE2* at the *Xba*I site. With unique flanking DNA and a 6 bp duplication of the *Xba*I site, the insert is 301 bp long, resulting in a frameshift that disrupts *ADE2*. As shown, repeats vary at the 14^th^ and 22^nd^ nucleotide. Type 1 repeats contain a G at position 14 and a C at position 22, Type 2 repeats contain G at both, Type 3 are C G, and Type 4 contain C at both. (B) Red/white color segregation in *ade2-h.75* strains. Strains were grown at 30° for 3 days, and then at room temperature for 4 days. The wild-type *ade2-min3* parent is DTK1188. Construction of the following strains is described above: *rad27*Δ (DTK1225), *end3*Δ (DTK1373), and *pkc1-4* (DTK1375). Note the presence in the *rad27*Δ strain of both blebs and sectors in the upper-right of the top colony. (C) Altered alleles of *ade2-h7.5* from 18 independent bleb isolates of DTK1225 (*ade2-h7.5 rad27*Δ) were sequenced. Altered allele structures are shown using the repeat number designations from the parental allele in Figure 3A. Repeats shown in gray have been added or modified. The location of deletions is illustrated as a gap in the repeat tract. For consistency, added repeats are shown to the right of the repeats they duplicate, although it is not possible to distinguish added and original repeats in the sequence.

The *ade2-min3* minisatellite tract consists of three identical tandem repeats. However, many human minisatellites, including the cancer-associated *HRAS1* minisatellite (Green and Krontiris 1993; Krontiris *et al.* 1993), contain repeat units with some sequence variation. Because sequence variation could limit homologous recombination between minisatellite repeats, different mechanisms could affect the stability of direct repeat minisatellite tract and variable repeat minisatellite tracts. To determine whether *RAD27*, *END3*, and *PKC1* regulate the stability of a variable repeat minisatellite tract, we utilized the previously described *ade2-h7.5* allele (Kelly *et al.* 2011), which contains seven and one-half repeats of 28 bp derived from the human *HRAS1* minisatellite inserted into the *ADE2* gene at the *Xba*I site (Figure 3A). The primary repeat sequence is 5′ GGCGTCCCCTGGA*G/C*AGAAGGG*G/C*GAGTGT 3′, with either a G or C at the 14^th^ and 22^nd^ positions, as indicated in italics.

We previously showed that loss of *ZRT1* destabilizes the *ade2-h7.5* minisatellite tract (Kelly *et al.* 2011). Similarly, the *ade2-h7.5 rad27*Δ strain exhibits a blebbing phenotype (Figure 3B). However, *end3*Δ and *pkc1-4* mutants do not display a blebbing phenotype in the *ade2-h7.5* background, raising the possibility that they might only regulate the stability of direct repeat minisatellite tracts. Whole-cell PCR across the minisatellite repeat tract was used to examine the nature of the tract alterations in independent bleb isolates of the *ade2-h.7 rad27*Δ strain. Of 112 alleles examined by PCR, 12 exhibited gain of two repeats, 7 exhibited loss of four repeats, and the majority, 92, exhibited loss of one repeat. A single allele was the same size as the unaltered *ade2-h7.5* tract; sequencing of this allele showed that it had a single nucleotide deletion, which reduces the size of the *HRAS1* minisatellite insert in *ADE2* from 301 bp to 300 bp and restores the correct reading frame (data not shown). Sequencing of 18 of the *ade2-h7.5 rad27*Δ minisatellite alleles examined by PCR revealed a wide range of events (Figure 3C). Of the 14 alleles that had lost a single repeat, 9 showed deletion of the fourth or fifth repeat. However, we saw examples of deletion of nearly every individual repeat in the seven and one-half repeat *ade2-h7.5* tract. We also obtained alleles exhibiting a loss of four repeats and a gain of two repeats. Finally, two strains had complex rearrangements indicative of multiple events. The first had a duplication of the second repeat coupled with a deletion of the fifth and sixth repeats. The second strain suffered two deletions of two repeats each. Both of these deletion events likely occurred between the two variable nucleotides in the repeat, leading to the formation of a novel repeat (indicated by gray repeats in Figure 3C, last row). The first deletion occurred between the G and C nucleotides of repeat 2 (at nucleotides 14 and 22, respectively) and the C and G nucleotides of repeat 4, forming a G G repeat (type 2), while the second deletion occurred between the G and G nucleotides of repeat 5 and the C and G nucleotides of repeat 7, forming a new type 2 repeat. The sequencing data indicate that very precise recombination events are occurring between minisatellite repeats in stationary phase cells.

## Discussion

Mutations in *END3*, *RAD27*, or *PKC1* stimulate alterations in a minisatellite tract while cells are in stationary phase; these alterations manifest as a blebbing phenotype in our assay system, allowing us to investigate aspects of post-mitotic genome maintenance using minisatellite tract alterations as an indicator of genome instability. Genetic analysis revealed that *END3*, *RAD27*, and *PKC1*, plus *ZRT1* and *ZAP1* (whose roles in minisatellite maintenance have been previously reported (Kelly *et al.* 2007), act in multiple independent pathways monitoring minisatellite stability during stationary phase. When the function of any of these pathways is disrupted, minisatellite alterations occur, with alterations being dependent on homologous recombination. Also, the human cancer-associated *HRAS1* minisatellite tract is destabilized during stationary phase by mutations in *RAD27* and *ZRT1*, but not *END3* or *PKC1*.

This is the first report of roles for *RAD27*, *PKC1*, and *END3* in post-mitotic genome maintenance. Rad27, a flap endonuclease, has a well-known role in Okazaki fragment processing and flap excision during long-patch base excision repair (BER) (Ayyagari *et al.* 2003; Wu and Wang 1999), and loss of *RAD27* has been shown to increase minisatellite alterations in actively dividing cells (Lopes *et al.* 2002; Maleki *et al.* 2002). In agreement with these data, we have observed a sectoring phenotype in *ade2-min3 rad27*Δ colonies, indicative of minisatellite repeat tract alterations in actively dividing cells, in addition to the strong stationary phase blebbing phenotype (Figure 1B). It is likely that a role for *RAD27* in post-mitotic genome stability has not been previously reported because other assay systems cannot easily distinguish between mitotic and post-mitotic events. Pkc1 is an essential protein kinase involved in signal transduction (Nishizuka 1992). *PKC1* mutants have a previously described hyper-recombination phenotype, which was interpreted as due to mitotic events (Huang and Symington 1994), but similarities between color segregation in the hyper-recombinant *PKC1* mutant (Huang and Symington 1994) and our previously reported blebbing strains (Kelly *et al.* 2007) prompted further investigation. We found that the *pkc1-4* mutation stimulates minisatellite alterations during stationary phase (Figure 1B); the lack of sectoring in the *ade2-min3 pkc1-4* strain indicates that *PKC1* does not regulate minisatellite stability during mitotic growth. The *END3* protein is involved in endocytosis (Benedetti *et al.* 1994) and has not previously been implicated in genome maintenance.

**Figure 4:**
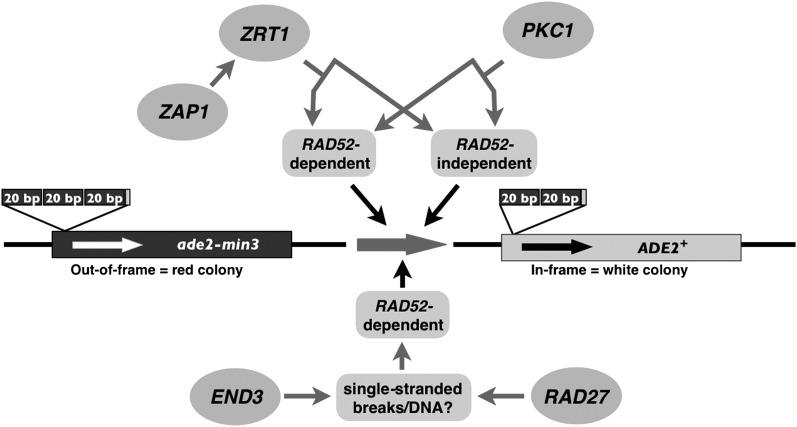
Model for pathways regulating *ade2-min3* minisatellite stability during stationary phase. Loss of components in multiple pathways can lead to *ade2-min3* minisatellite alterations. In the first pathway, loss of *ZRT1* or *ZAP1* stimulates loss of one *ade2-min3* repeat unit via both *RAD52*-dependent and *RAD52*-independent recombination (Kelly *et al.* 2007). In a second pathway, mutation of *PKC1* stimulates loss of one *ade2-min3* repeat unit via both *RAD52*-dependent and *RAD52*-independent recombination. A third independent pathway is represented by *END3*, loss of which stimulates deletion of one *ade2-min3* repeat unit via *RAD52*-dependent recombination. This deletion may be due to formation of ssDNA as a consequence of oxidative DNA damage. Alternatively, *END3* could be acting in the *RAD52*-dependent portions of the *ZRT1* and *PKC1* pathways (not shown). In the final pathway, loss of *RAD27* stimulates both loss of one and gain of two *ade2-min3* repeats. These minisatellite alterations may be due to formation of ssDNA when *RAD27*-dependent DNA flap removal does not occur properly. *RAD27*-dependent minisatellite alterations during mitotic growth require *RAD52*, so it is possible that stationary phase minisatellite alterations in this pathway also occur via *RAD52*-dependent recombination.

Genetic analysis with blebbing mutants identified in this study and previous work (Kelly *et al.* 2007) demonstrate that up to four pathways monitor minisatellite stability during stationary phase (Figure 4). One interpretation of our data is that each acts in an independent pathway: the first pathway is represented by *ZRT1* and *ZAP1*, the second by *RAD27*, the third by *END3*, and the fourth by *PKC1*. While these pathways are at least partially independent, one common factor may be the involvement of single-stranded DNA (as shown in Figure 4). A second possibility is that *END3* may be acting in the *ZRT1* and the *PKC1* pathways as a component of the *RAD52*-dependent repair activity, rather than representing an independent pathway.

The *ZRT1*/*ZAP1* pathway has been characterized in detail elsewhere (Kelly *et al.* 2007, 2011). Loss of the *RAD27* pathway causes an increase in stationary phase minisatellite alterations, which include both gain and loss of repeat units. Rad27 processes DNA flaps during Okazaki fragment maturation and long-patch BER in actively growing cells (Ayyagari *et al.* 2003; Wu and Wang 1999), but faulty Okazaki fragment processing is not a likely cause of minisatellite alterations during stationary phase, as bulk DNA synthesis does not occur in post-mitotic cells. However, replication during DNA repair events, such as long-patch BER, does occur in post-mitotic cells (Barzilai *et al.* 2008), and in the absence of *RAD27*, unprocessed DNA flaps could be resolved by homologous recombination. If such an event occurred within the minisatellite tract, misalignment of repeat units during recombination could account for the stationary phase minisatellite alterations seen in the *RAD27* mutant. In addition, absence of Rad27 may cause an increase in ssDNA due to flap processing failure; this ssDNA may accumulate breaks or suffer repeat misalignment during repair synthesis, leading to changes in repeat number.

Loss of the *PKC1*-dependent pathway results in a significant increase in minisatellite alterations that occur via both *RAD52*-dependent and *RAD52*-independent recombination, but not NHEJ. *PKC1* is known to have a role in stationary phase entry, downstream of TOR inactivation (Gray *et al.* 2004). Mutants defective for *PKC1* show a substantial decrease in viability when starved (Krause and Gray 2002). Some *PKC1* mutant cells fail to enter stationary phase when stressed, continuing to grow and replicate their DNA in spite of severely limited resources. DNA synthesis under these circumstances is likely to lead to ssDNA formation, replication fork stalling, and collapse, which can serve as a substrate for homologous recombination (Branzei and Foiani 2005). If *pkc1-4 ade2-min3* cells that do enter stationary phase also exhibit DNA replication control abnormalities, ssDNA formation followed by fork stalling might occur within the minisatellite tract. Subsequent misalignment of the repeat units during recombination might explain the stationary phase blebbing phenotype and minisatellite alterations seen in the *PKC1* mutant. However, few of the downstream effectors of the Pkc1 kinase have been identified, so it is possible that this protein may play an as yet unknown role in minisatellite stability during stationary phase.

Stationary phase minisatellite alterations are also increased in cells with a deletion of *END3*. Minisatellite alterations in an *end3*Δ mutant arise through *RAD52*-dependent recombination, unlike the *ZRT1* and *PKC1* pathways. Although *END3* has no previously described role in genome maintenance, *end3*Δ mutants display a stationary phase–specific increase in ROS production (Gourlay and Ayscough 2006). Consistent with this result, our *ade2-min3 end3*Δ and *end3-1* strains display an elevated level of ROS production during stationary phase (Figure 2). ROS production is reduced in an *END3* mutant with a *RAS2* deletion, a change that is concomitant with a decrease in blebbing. ROS can cause many types of DNA damage, including ssDNA formation and DNA breaks (Jackson and Loeb 2001). If ROS-triggered DNA breaks occur within the minisatellite, misalignment of repeat units during repair by homologous recombination could account for *RAD52*-dependent minisatellite alterations in stationary phase *END3* mutants.

Mutations in *RAD27*, *END3*, and *PKC1* all destabilize the *ade2-min3* minisatellite tract, but only loss of *RAD27* destabilizes the *HRAS1* minisatellite repeats in *ade2-h7.5* (Figure 3B). The *ade2-min3* tract is composed of identical repeat units (Figure 1A), but *ade2-h7.5* is composed of repeat units whose sequence varies at two nucleotides (Figure 3A). While minisatellite alterations are more frequent in the *ade2-min3 rad27*Δ strain (32.0 blebs/colony; Table 2) than in the *ade2-h7.5 rad27*Δ strain (5.0 blebs/colony; Figure 3B), both mutant strains show a similar fold increase compared with the parental wild-type strain (9-fold for *ade2-min3* and 7-fold for *ade2-h7.5*). Thus, the *HRAS1* minisatellite tract is more stable than the *ade2-min3* tract, but the effect of *RAD27* loss is approximately the same for both minisatellites. In contrast, *END3* and *PKC1* mutations affect the stability of the *ade2-min3* repeat tract but not the *ade2-h7.5* tract. This result indicates that pathways monitoring *ade2-h7.5* stability may differ from those monitoring *ade2-min3* stability, with the influence of *END3* and *PKC1* being limited to direct repeat minisatellites.

Almost all of the altered alleles in an *ade2-h7.5 rad27*Δ strain exhibit tract expansions or contractions; less than 1% of minisatellite tracts examined by PCR were similar in size to the parental *ade2-h7.5* allele. Due to the sequence variation between repeats in *ade2-h7.5* strains, we are able to determine the exact nature of the alterations that give rise to blebs. We sequenced 18 altered alleles (Figure 3C). Most alleles are the result of one deletion or duplication event in the original repeat tract, likely occurring via homologous recombination, with a strong bias toward alteration of the center repeats. However, two alleles (5′-411233-3′ and 5′-422-3′) are more complex, indicating that some tract alterations in the *ade2-h7.5 rad27*Δ mutant may derive from more than one event.

Many mechanisms for genome maintenance are conserved between yeast and humans (Taylor and Lehmann 1998). Therefore, it is likely that our results will be applicable to post-mitotic genome maintenance in human cells, especially as both *RAD27* and *PKC1* have human homologs (Liu *et al.* 2004; Nishizuka 1992). We have shown that *RAD27* regulates the stability of the human cancer-associated *HRAS1* minisatellite. Our results indicate *PKC1* and *END3* may regulate the stability of direct repeat minisatellites only. As some minisatellites that are correlated with human disease are composed of direct repeats, such as the minisatellite associated with progressive myoclonus epilepsy (Lafreniere *et al.* 1997), our results establish a mechanistic link between factors controlling direct repeat stability and human disease.
